# Adjacent Infarction in the Lenticulostriate Artery Territory Associated With Hypertensive Putaminal Hemorrhage: A Case Report

**DOI:** 10.7759/cureus.113433

**Published:** 2026-07-27

**Authors:** Junpei Nagasawa, Eiko Fujimoto, Junya Ebina, Takehisa Hirayama

**Affiliations:** 1 Neurology, Toho University Faculty of Medicine, Tokyo, JPN

**Keywords:** cerebral infarction, diffusion-weighted imaging, hemorrhagic transformation, lenticulostriate artery, putaminal hemorrhage

## Abstract

Acute ischemic lesions are occasionally detected in patients with intracerebral hemorrhage, but they are usually remote from the hematoma. Acute infarction adjacent to hypertensive putaminal hemorrhage is extremely rare. We report a 52-year-old man with untreated hypertension who presented with right upper extremity weakness, gait unsteadiness, and left temporal headache. Computed tomography showed a left putaminal hematoma with surrounding edema and a subtle adjacent hypoattenuating area. Magnetic resonance imaging demonstrated an acute infarction adjacent to the superomedial aspect of the hematoma, extending toward the left corona radiata within the lenticulostriate artery territory. No major arterial stenosis, vascular malformation, cardioembolic source, coagulopathy, or vasculitis was identified. His symptoms gradually improved with antihypertensive therapy. The mechanism remains uncertain, but hematoma-related local compression or hypoperfusion and concomitant hypertensive small vessel pathology may have contributed to the adjacent infarction. This case highlights the importance of differentiating hematoma-associated adjacent infarction from hemorrhagic transformation of ischemic stroke.

## Introduction

Hypertensive putaminal hemorrhage is one of the most common forms of spontaneous intracerebral hemorrhage [1,2]. These hemorrhages typically result from rupture of the lenticulostriate arteries, small deep perforating vessels that are particularly vulnerable to chronic hypertensive changes such as lipohyalinosis and microaneurysm formation. Consequently, the lenticulostriate territory is susceptible to both hemorrhagic and ischemic injury related to hypertensive small vessel disease. Acute ischemic lesions are occasionally detected on diffusion-weighted imaging in patients with intracerebral hemorrhage; however, these lesions are usually located remote from the hematoma and are considered to be associated with small vessel disease, acute blood pressure reduction, or hemodynamic alterations [3].

In contrast, acute ischemic infarction developing immediately adjacent to a putaminal hematoma within the same lenticulostriate artery territory is extremely rare. Proposed mechanisms include hematoma-related local compression or hypoperfusion, as well as concomitant hemorrhagic and ischemic injury within the same perforating arterial territory [4,5].

Here, we report a patient with hypertensive putaminal hemorrhage accompanied by an adjacent acute infarction in the lenticulostriate artery territory. Accurate recognition of this rare entity is clinically important because it may influence the diagnostic evaluation and subsequent management and prevent misinterpretation as hemorrhagic transformation of ischemic stroke. We discuss the radiological findings, possible mechanisms, and differential diagnosis of hemorrhagic transformation of ischemic stroke.

## Case presentation

A 52-year-old man with untreated hypertension was referred to our hospital for evaluation of right upper extremity weakness, gait unsteadiness, and left temporal headache. He had been noted to have hypertension two years earlier but had not received antihypertensive treatment. He had no other significant medical history. He had never smoked and drank approximately 500 mL of beer every evening.

**Figure 1:**
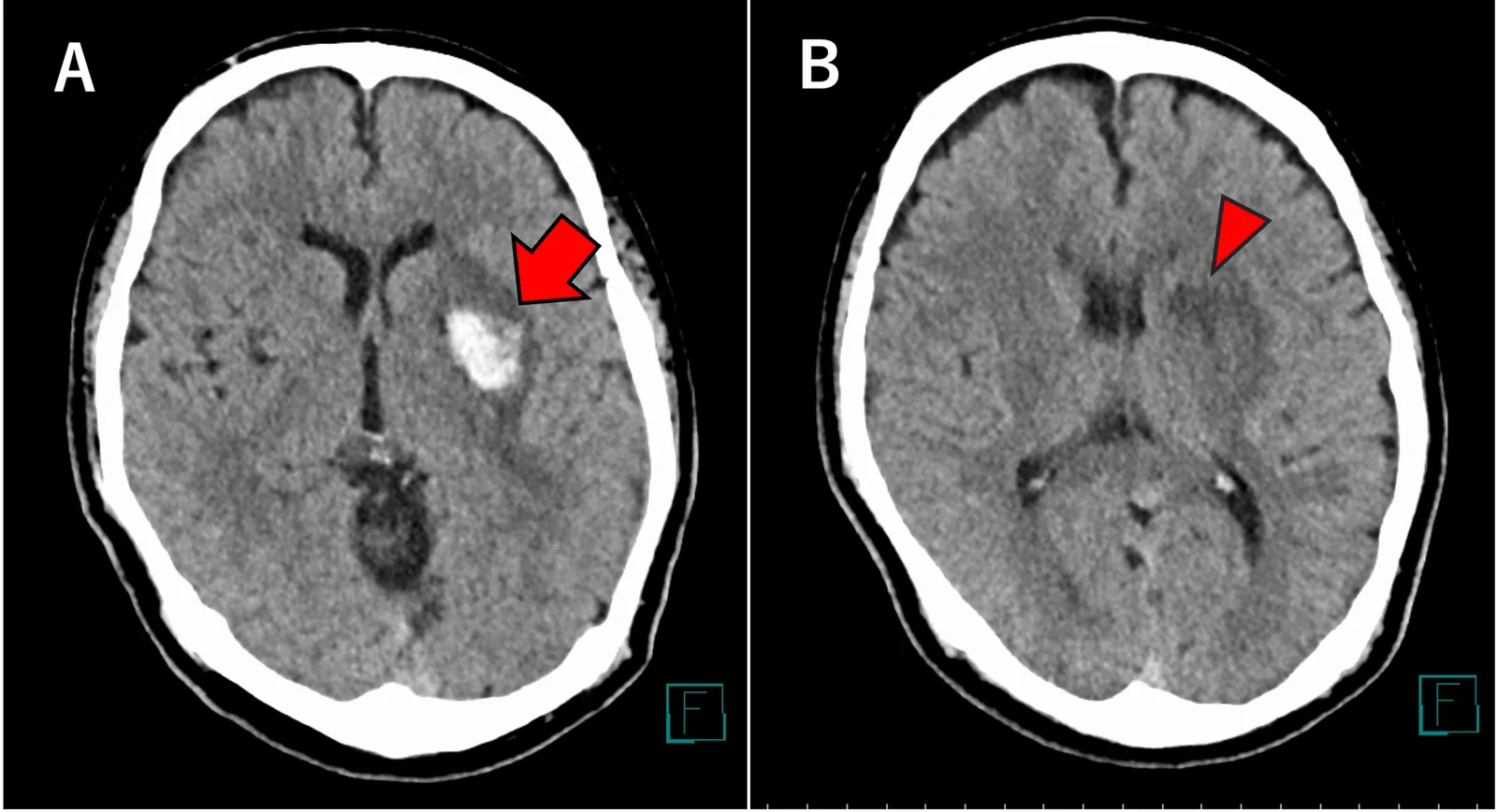
Initial non-contrast computed tomography of the head. (A) Axial computed tomography showed a left putaminal hematoma with surrounding edema (arrow). (B) A slightly more cranial slice showed a subtle hypoattenuating area adjacent to the hematoma, extending toward the left corona radiata within the lenticulostriate artery territory (arrowhead).

At approximately 1:00 PM, he suddenly developed these symptoms. As they persisted, he visited a local clinic four days after symptom onset. His blood pressure was 204/140 mmHg, and he was referred to our hospital on the same day. On arrival at our hospital, his right upper extremity weakness had nearly resolved. Mild aphasia was noted, and his National Institutes of Health Stroke Scale score was 1. Non-contrast computed tomography (CT) of the head revealed a left putaminal hematoma with surrounding edema. The hematoma volume was approximately 6.4 mL, calculated using the ABC/2 method. In addition, a subtle hypoattenuating area was observed adjacent to the superomedial aspect of the hematoma, extending toward the left corona radiata (Figures 1A-1B).

**Figure 2:**
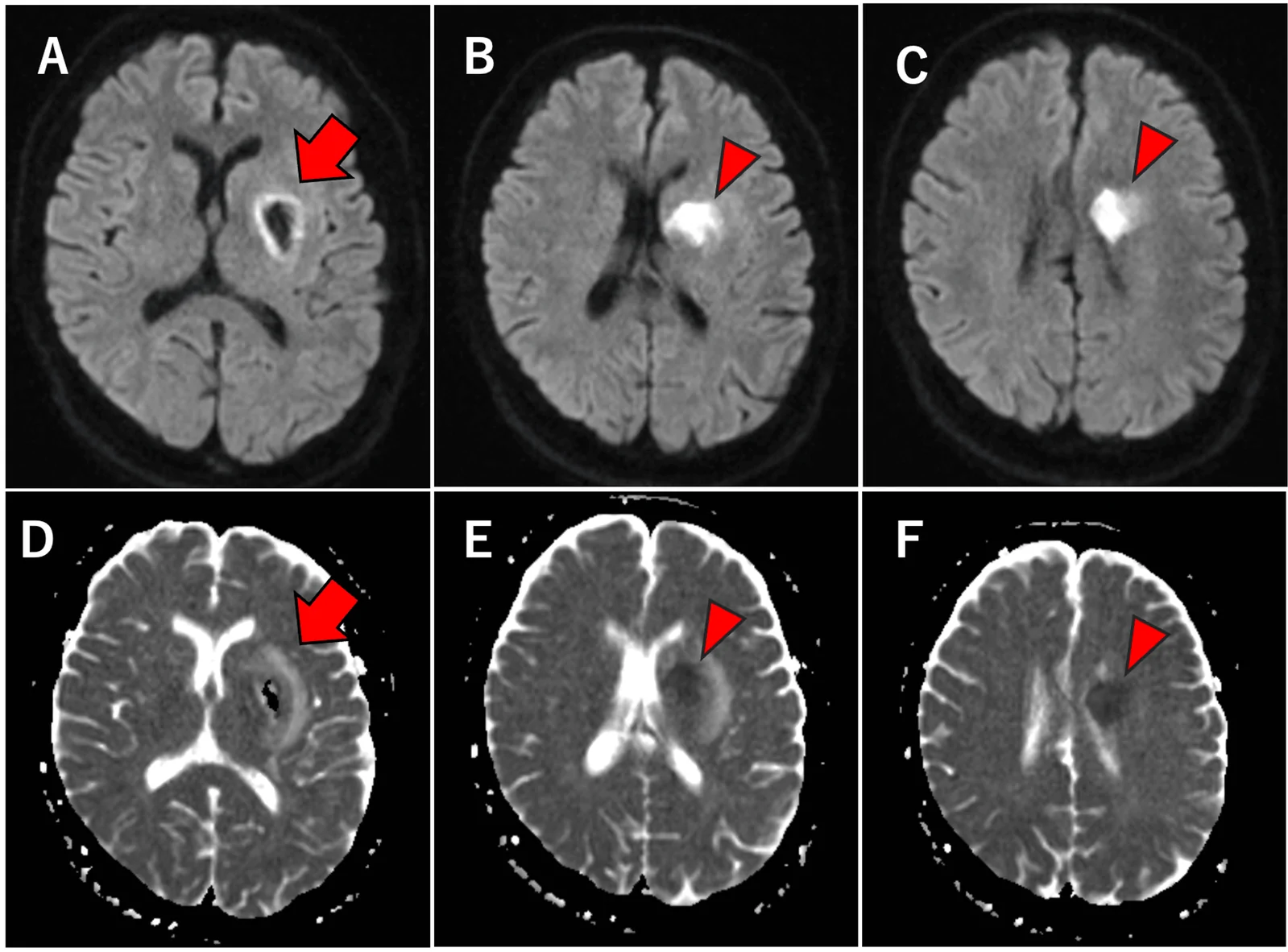
Magnetic resonance imaging findings. (A) Axial diffusion-weighted imaging showed a left putaminal hematoma characterized by a central area of low signal intensity - due to magnetic susceptibility effects - surrounded by a hyperintense rim of perihematomal edema (arrow). (B, C) More cranial slices demonstrated a hyperintense lesion adjacent to the superomedial aspect of the hematoma, extending toward the left corona radiata within the lenticulostriate artery territory (arrowheads). The lesion was consistent with acute infarction. (D, E, F) Corresponding apparent diffusion coefficient maps showed high signal intensity in the perihematomal region, consistent with edema (arrow), whereas the adjacent lesion extending toward the corona radiata showed low signal intensity, indicating restricted diffusion and acute infarction (arrowheads).

Brain magnetic resonance imaging (MRI) was subsequently performed. Consistent with the CT findings, MRI showed a left putaminal hematoma with surrounding edema. Diffusion-weighted imaging revealed a hyperintense lesion adjacent to the superomedial aspect of the hematoma, extending toward the left corona radiata, with corresponding low signal intensity on the apparent diffusion coefficient (ADC) map, indicating acute infarction in the lenticulostriate artery territory (Figures 2A-2F). The ischemic lesion was considered distinct from perihematomal edema because the perihematomal region showed increased diffusion (high signal intensity) on the ADC map, whereas the adjacent lesion showed low ADC signal intensity, consistent with true restricted diffusion (Figures 2D-2F).

Magnetic resonance angiography, CT angiography, and carotid ultrasonography showed no stenosis or occlusion of the major intracranial or extracranial arteries. No vascular abnormalities, including arterial dissection, aneurysm, arteriovenous malformation, or dural arteriovenous fistula, were identified. Holter electrocardiography and transthoracic echocardiography showed no atrial fibrillation, left atrial enlargement, or other high-risk sources of cardioembolism.

Based on the presence of severe, untreated hypertension and the location of the hematoma, the patient was diagnosed with hypertensive putaminal hemorrhage. Continuous intravenous nicardipine was initiated for blood pressure control, with a target systolic blood pressure of <140 mmHg. His neurological symptoms gradually improved after admission without further neurological deterioration. He was discharged home after clinical stabilization with a modified Rankin Scale score of 1, indicating functional independence.

## Discussion

In the present case, an acute infarction in the lenticulostriate artery territory was identified adjacent to a hypertensive putaminal hematoma. Although remote ischemic lesions are occasionally observed in patients with intracerebral hemorrhage [6], infarction immediately adjacent to a putaminal hematoma is extremely rare. Following a review of the literature, we identified only two previously reported cases describing acute infarction adjacent to hypertensive putaminal hemorrhage. Therefore, to the best of our knowledge, the present case represents the third reported case of acute infarction adjacent to hypertensive putaminal hemorrhage.

Yasuda et al. reported two patients who developed perforator infarction adjacent to hypertensive intracerebral hemorrhage several days after the onset of hemorrhage and proposed hematoma-related local compression or hypoperfusion as the underlying mechanism [5]. Subsequently, Inomata et al. reported a patient who developed corona radiata infarction several days after putaminal hemorrhage within the same lenticulostriate arterial territory, suggesting involvement of the same perforating arterial territory [4].

Although the temporal sequence could not be determined because MRI was performed four days after symptom onset, the spatial relationship between the putaminal hematoma and adjacent infarction in the lenticulostriate artery territory was remarkably similar to that observed in the previously reported cases.

Several mechanisms may explain the development of infarction adjacent to putaminal hemorrhage in the lenticulostriate artery territory. First, the mass effect of the hematoma may have increased local interstitial tissue pressure, resulting in mechanical compression of adjacent lenticulostriate perforating arteries and subsequent microcirculatory impairment in the surrounding tissue. This mechanism is consistent with the hypothesis proposed in previous reports of delayed perforator infarction adjacent to hypertensive intracerebral hemorrhage.

Second, underlying hypertensive small vessel disease may have contributed to both hemorrhagic and ischemic injury within the lenticulostriate artery territory. Pathological changes, such as lipohyalinosis or fibrinoid degeneration, can lead to either rupture or occlusion of perforating arteries. Therefore, concomitant rupture and occlusion within the same perforating arterial system may explain the coexistence of putaminal hemorrhage and adjacent infarction.

Acute blood pressure reduction has also been proposed as a possible contributor to ischemic lesions after intracerebral hemorrhage. However, this mechanism was considered unlikely in the present case because the infarction was already evident on the initial MRI obtained before antihypertensive treatment was initiated at our hospital. Therefore, local mechanisms related to the hematoma itself or to the pathology of the lenticulostriate artery territory may better explain the adjacent infarction in this patient.

Nevertheless, hemorrhagic transformation of an ischemic infarction cannot be completely excluded. Because MRI was performed four days after symptom onset, the temporal sequence between hemorrhage and infarction could not be determined. However, the typical location and morphology of the putaminal hematoma, together with the extension of the infarction superiorly and medially within the lenticulostriate artery territory rather than surrounding the hematoma, may favor hematoma-associated adjacent infarction over hemorrhagic transformation.

This report has several limitations. First, an MRI was performed four days after symptom onset; therefore, the temporal sequence between hemorrhage and infarction could not be determined.

Second, direct hemodynamic evidence of hematoma-related compression or hypoperfusion within the lenticulostriate artery territory was unavailable due to the lack of acute perfusion-weighted MRI or advanced microvascular imaging, which might have provided additional information regarding the underlying mechanism. Third, pathological confirmation of the underlying hypertensive small vessel disease was not obtained. Therefore, the exact mechanism of the adjacent infarction remains uncertain.

## Conclusions

Acute infarction adjacent to hypertensive putaminal hemorrhage is extremely rare. Although the precise mechanism remains uncertain, hematoma-associated infarction in the lenticulostriate artery territory should be considered when acute ischemic lesions are detected adjacent to a putaminal hematoma, while carefully differentiating it from hemorrhagic transformation of ischemic stroke. Further accumulation of similar cases and larger studies is needed to better elucidate the underlying mechanism of this rare presentation.
